# Dielectric and Structural Properties of the Hybrid Material Polyvinylidene Fluoride-Bacterial Nanocellulose-Based Composite

**DOI:** 10.3390/polym15204080

**Published:** 2023-10-13

**Authors:** Aleksandra Janićijević, Suzana Filipović, Aleksandra Sknepnek, Branislav Vlahović, Nenad Đorđević, Danijela Kovacević, Miljana Mirković, Ivan Petronijević, Predrag Zivković, Jelena Rogan, Vladimir B. Pavlović

**Affiliations:** 1 The Academy of Applied Technical Studies Belgrade, 11000 Belgrade, Serbia; djordjevicn@atssb.edu.rs (N.Đ.); dkovacevic@atssb.edu.rs (D.K.); 2 Institute of Technical Sciences of Serbian Academy of Sciences and Arts, 11000 Belgrade, Serbia; 3 Faculty of Agriculture, University of Belgrade, 11000 Belgrade, Serbia; aleksandras@agrif.bg.ac.rs (A.S.); vladimirboskopavlovic@gmail.com (V.B.P.); 4 Department of Mathematics and Physics, North Carolina Central University, Durham, NC 27707, USA; vlahovic@nccu.edu; 5 NASA University Research Center for Aerospace Device Research and Education, NSF Center of Research Excellence in Science, Technology Computational Center for Fundamental and Applied Science and Education, Durham, NC 27707, USA; 6 Department of Material Science, “VINČA” Institute of Nuclear Sciences-National Institute of the Republic of Serbia, University of Belgrade, 11000 Belgrade, Serbia; miljanam@vin.bg.ac.rs; 7 Faculty of Physics, University of Belgrade, 11000 Belgrade, Serbia; ivanpetronijevic@ff.bg.ac.rs; 8 Faculty of Technology and Metallurgy, University of Belgrade, 11000 Belgrade, Serbia; peca@tmf.bg.ac.rs (P.Z.); rogan@tmf.bg.ac.rs (J.R.)

**Keywords:** dielectric properties, multiferroic, laminate composite material, bacterial nanocellulose (BNC), PVDF

## Abstract

In the search for environmentally friendly materials with a wide range of properties, polymer composites have emerged as a promising alternative due to their multifunctional properties. This study focuses on the synthesis of composite materials consisting of four components: bacterial nanocellulose (BNC) modified with magnetic Fe_3_O_4_, and a mixture of BaTiO_3_ (BT) and polyvinylidene fluoride (PVDF). The BT powder was mechanically activated prior to mixing with PVDF. The influence of BT mechanical activation and BNC with magnetic particles on the PVDF matrix was investigated. The obtained composite films’ structural characteristics, morphology, and dielectric properties are presented. This research provides insights into the relationship between mechanical activation of the filler and structural and dielectric properties in the PVDF/BT/BNC/Fe_3_O_4_ system, creating the way for the development of materials with a wide range of diverse properties that support the concept of green technologies.

## 1. Introduction

Hybrid materials are a broad research field, focusing on the synthesis of new and innovative materials [1]. These materials offer unique properties that bring numerous benefits to various aspects of human life. Key areas of interest include the development of sustainable biosensors, active and intelligent packaging, renewable energy storage materials, batteries, nanogenerators, and microchips [2,3,4]. However, creating such materials poses a challenge due to the need to combine different material classes with the aim to develop a flexible, biodegradable, biocompatible material with multiferroic properties. In this context, it is crucial to consider the environmental impact of materials while meeting practical criteria. Traditional packaging materials mainly are not sustainable, thus requiring the implementation of “green” technologies and the use of environmentally friendly materials. The material that stands out for its unique characteristics is polyvinylidene fluoride (PVDF). PVDF is a polymer widely used as a matrix material in various applications. It exhibits exceptional electroactive properties and can exist in different crystalline phases, with the beta phase being particularly desirable due to its superior electrical characteristics [5]. The activation of the beta phase in PVDF can be achieved through various processes and treatments, such as temperature changes, deformation, particle size and shape, exposure to air, and the addition of appropriate fillers [6,7,8,9,10]. Research has shown that incorporating ceramic fillers, especially perovskite ceramic materials, is one of the most effective ways to activate the beta phase of PVDF [11,12,13]. A filler that meets all criteria is barium titanate (BaTiO_3_), known for its high die lectric constant and ferroelectric properties [14,15]. When BaTiO3 is incorporated into the PVDF matrix, it enhances the electrical polarization and improves the electroactive properties of the material [16,17]. Prior studies [18,19] also demonstrated that mechanically activating BaTiO3 particles before combining with PVDF can encourage β-phase crystallization in the matrix. This involves reducing particle size without altering their ferroelectric tetragonal structure [19]. As a result, the filler addition increases dielectric permittivity and promotes crystallization of polar β-phase crystals, achieving the desired properties with lower filler concentrations.

On the other hand, bacterial cellulose has also gained significant attention in hybrid materials research [20,21]. Bacterial cellulose is a biopolymer produced through the fermentation of bacteria such as *Gluconacetobacter xylinus*. It possesses high purity, mechanical strength, water resistance, and biocompatibility [22]. These properties make bacterial cellulose attractive for various applications such as membranes, biofilters, cell culture media, and even in skin and bone tissue repair [23,24,25]. Combining bacterial cellulose with other materials like PVDF allows the creation of hybrid materials with improved mechanical and electrical properties and the ability to tailor their characteristics for specific applications [26,27]. However, hybrid materials that combine PVDF with fillers such as BaTiO_3_ and bacterial cellulose hold great promise for the development of advanced materials with enhanced mechanical, electrical, and biological properties. In addition to the combination of PVDF, BaTiO_3_, and bacterial cellulose, the possibility of modifying bacterial cellulose with magnetite (Fe_3_O_4_) is worth mentioning. Modifying bacterial cellulose with magnetite allows the creation of a hybrid material with magnetic properties.

Very important features for intelligent packaging are dielectric properties. Hybrid materials developed for sensor applications can change their dielectric permittivity in contact with humidity or by variation in temperature. Such change can be used for monitoring the transportation and storage of products [28]. The relative dielectric constant (ε′) has a significant physical implication as it delineates a material’s capacity to store and retain electrical energy within its structure. Conversely, dielectric losses include the dissipation of energy within the material itself. The loss tangent, denoted as tan δ, represents the ratio of these two physical quantities, namely the relationship between dielectric losses (ε″) and relative dielectric constant (ε′) [29]. An increasing number of recent studies are focused on the synthesis of layered composites to achieve high dielectric constants with minimal losses, while also ensuring the preservation of composite flexibility [30,31,32].

Taking into account a variety of the properties that the BNC, PVDF, BT, and Fe_3_O_4_ possess, they represent a potentially brilliant combination. The idea of this research was to synthesize and investigate a laminar multifunctional composite, where the first layer, Fe_3_O_4_-modified BNC, will achieve connection with the PVDF/BT layer by hot press. So, the base or matrix of the synthesized hybrid material is PVDF, while BNC with magnetite acts as one filler, while BT acts as the other filler. Further influence of the mechanical activation of BT on PVDF phase composition was linked with obtained dielectric properties of the hybrid composite. BT was mechanically activated for 5, 10, and 20 min. The synthesized films were characterized by SEM-EDS, XRD, PSA, and DS methods.

## 2. Materials and Methods

In this research, PVDF powder with an average molecular weight of Mw~534,000 (Sigma-Aldrich, St. Louis, MO, USA) and BaTiO_3_ (BT) 99.5%, <2 μm (Sigma-Aldrich, St. Louis, MO, USA) were used. An acetic acid bacteria (*Komagataeibacter* sp.) and 0.1 M NaOH solution was used to synthesize and subsequently purify bacterial nanocellulose. FeSO_4_x7H_2_O (Acros Organics, Waltham, MA, USA), FeCl_3_x6H_2_O (Fisher Chemical, Waltham, MA, USA), and NH_3_(aq) (Sigma-Aldrich, St. Louis, MO, USA) solutions were used for further modifi cation of the bacterial nanocellulose. Ethanol from Merck was utilized as a homogenizing medium for PVDF and BT powders.

### 2.1. Preparation of BNC/Fe_3_O_4_

Initially, the synthesis of bacterial nanocellulose (BNC) was performed via fermentation of acetic acid bacteria isolated from Kombucha [33]. The hydrogels were grown in a YPM broth (yeast extract 5 g/L, peptone 3 g/L, and mannitol 25 g/L) for 7 days at 25 °C under static conditions. The resulting BNC films were then purified and submerged in a solution containing diluted iron salts (FeSO_4_x7H_2_O + FeCl_3_x6H_2_O) at a molar ratio of 2:1. Hydrogels were sonicated for 30 min. Subsequently, precipitation of magnetite was achieved by the addition of NH_3_(aq) to bring the pH to approximately 12. The films were then sonicated for an additional 45 min. After washing with distilled water to reach a neutral pH, films were dried in an oven at 40 °C for 24 h.

### 2.2. Preparation of Multi-Component Composites PVDF/BaTiO_3_/BNC/Fe_3_O_4_

Commercial BaTiO_3_ was mechanically activated in a Retsch PM100 planetary mill using a zirconium oxide jar and balls. Small 5 mm diameter balls were used. Mechanical activation was carried out at intervals of 5, 10, and 20 min. The mass ratio of the ball/sample was 20:1 and the rotation speed was 400 rotations per minute. The obtained powders were labeled as BT5, BT10, and BT20. The synthesis of the PVDF/BT layer was carried out in two steps. First, 5 wt% of the mechanically activated BT was homogenized with PVDF in ethanol using an ultrasonic bath for 30 min. Ethanol was selected as the medium because neither component is soluble in it. The suspensions were centrifuged on a UNIVERSAL320 R device for 10 min and then left in an oven at 80 °C for 24 h to completely evaporate the ethanol. The powder mixtures were labeled as PVDF, PVDF/BT0, PVDF/BT5, PVDF/BT10, or PVDF/BT20, where PVDF was pure polymer without BT addition and the rest of the samples contained non-activated and activated BT powders for 5–20 min. The multilayer composite of the PVDF/BT and BNC/Fe_3_O_4_ films were prepared by hot pressing. The SERVITECpolystat200T press was used for this purpose. A 1.5 g of the previously prepared homogeneous PVDF-based mixtures was evenly distributed on the dry BNC/Fe_3_O_4_ film.

The samples were placed on square steel molds, 10 × 10 cm^2^ in dimension, which were coated with Teflon to avoid sticking. The pressing was carried out under the following conditions: an initial heating of the press jaws for 6 min at a temperature of 185 °C, followed by a two-stage pressure ramping. At the beginning of the process, a pressure of 20 bars was applied to the steel mold for 1 min, followed by a 2 min hot pressing under 200 bars. In this way, five two-layer films were obtained, and the resulting films were labeled as follows: PVDF/BNC/Fe_3_O_4_, PVDF/BT0/BNC/Fe_3_O_4_, PVDF/BT5/BNC/Fe_3_O_4_, PVDF/BT10/BNC/Fe_3_O_4_, and PVDF/BT20/BNC/Fe_3_O_4_. We kept the thickness of the films constant. All two-layer samples were (0.22 ± 0.01) mm thick, while single BNC/Fe_3_O_4_ layers were (0.9 ± 0.01) mm thick, and single PVDF/BT layers had a thickness of (0.13 ± 0.01) mm.

### 2.3. Characterization Techniques

The particle size distributions (PSA) of non-activated and activated BT samples, pure PVDF, and PVDF/BT powder mixtures were analyzed using laser diffraction (Mastersizer 2000, Malvern Instruments, Malvern, UK). XRD characterization was performed on a Rigaku Ultima IV diffractometer using CuKα1,2 radiation and a D/TeX Ultra high-speed detector. For BT powder samples and BNC/Fe_3_O_4_/PVDF/BT film samples, the range was 10° to 90° 2*θ*. The structural parameters (crystallite sizes and strains) were determined using the RIR method and PDXL2 software with PDF2 Release 2012 database. SEM (Scanning Electron Microscopy) and EDS (Energy-dispersive X-ray Spectroscopy) analysis are techniques that are used to investigate the morphology and microstructure of synthetic films. The samples were coated with gold for 100 s at 30 mA using a Bal-tec SCD 005 Sputter coater. After preparation, samples were imaged on a JEOL JSM-6390LV device equipped with software for EDS analysis, Oxford Instruments X-MaxN. Dielectric spectroscopy (DS) measurements were performed using Hameg 8118 and Agilent 4285A RCL bridges. The frequency dependence of the relative dielectric constant (ε′) and loss tangent (tan δ) was recorded in the 20 Hz to 9 MHz frequency range using a sinusoidal excitation voltage of 1.5 V and a parallel capacitive measurement model (*Cp*). Measurements were conducted at normal atmospheric pressure, 23 °C temperature, and 40% relative humidity.

## 3. Results and Discussion

**Figure 1:**
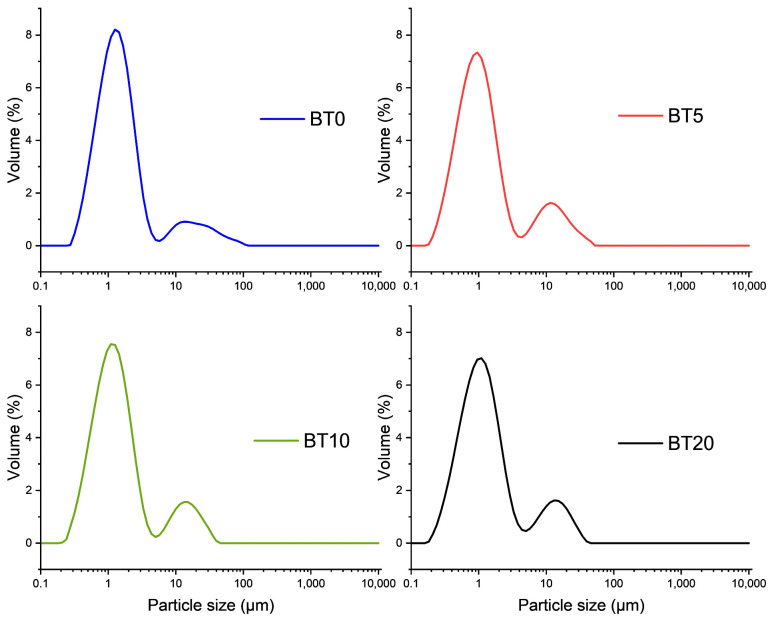
Dependency of volume percentage on particle size for samples BT0, BT5, BT10, and BT20.

Mechanical activation as a process for the reduction in particle size and increase in its reactivity and surface activity has been widely used [34,35]. During milling, particles are subjected to intense contact between each other as well as between particles and milling media and jar walls. That leads to plastic deformations, particle and crystallite size reduction, and the formation of defective surfaces, which is highly active [36]. Figure 1 illustrates the logarithmic distribution of particle sizes for the commercial BT and activated powders.

The particle distribution is expressed by the following derived diameters d(0.1), d(0.5), and d(0.9) presented in Table 1. The graphs display a bimodal distribution, with two distinct fractions. The dominant fraction reveals that particles of the commercial barium-titanate have a diameter of around 1.458 μm. With mechanical activation, the peaks of BT5, BT10, and BT20 shift to the left, indicating a decrease in particle size. The median mass diameter of the volume distribution d(0.5) for BT5 was 1.115 μm, while for samples

BT10 and BT20, these values were 1.336 μm and 1.244 μm, respectively. The second fraction extends in the range of particle diameters from 10 to 100 μm, and the volume percentage compared to the first fraction indicates a smaller value. This fraction originates from agglomerates present in the commercial BT powder. Mechanical activation for 5–20 min was not sufficient for complete agglomerate destruction but sufficient for significant reduction in the biggest ones that were approximately 100 μm in size. The span parameter, which refers to the width of the particle distribution, indicates that with the activation time of BT, the distribution widens. The value of the span for BT is 6.904, while for other samples BT5, BT10, and BT20, this value increases (Table 1).

**Figure 2:**
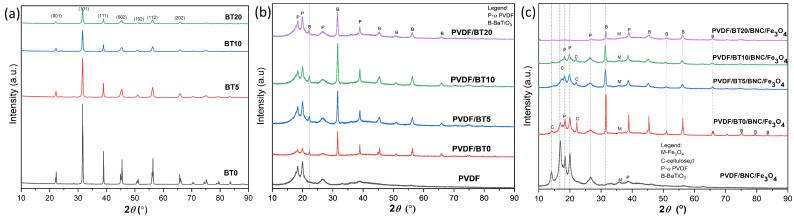
(**a**) XRD patterns of the BT powders; (**b**) XRD patterns of the PVDF/BT mixed powders, (**c**) diffractograms of two-layer hybrid composite films (PVDF/BT side of the films).

According to the identified crystalline peaks presented on XRD patterns of the BT powders, Figure 2a, the tetragonal crystal structure of the BT particles was confirmed for non-activated powder and remains unaltered following the process of mechanical activation. Due to a rise in structural disorder, the mechanically activated particles display lower peak intensities of reflections with the diffraction profile broadening. The diffraction of the BT powder, shown in Figure 2a, also shows that with a longer period of grinding, there is a significant reduction in the intensity of the strongest reflections (101, 111, and 002) as well as the widening of these reflections. Due to the grinding process, it is evident that the crystallite sizes are decreasing, as well as the grains. This decrease in crystallite sizes and grain sizes is a result of the mechanical activation (Table 2). Additionally, the reduction in the intensity of the strongest reflections and the widening of these reflections indicates a reduction in the degree of crystallinity of the obtained material.

Although this effect is evident in the BaTiO_3_ diffraction profiles (Figure 2a), it is less pronounced in the BaTiO_3_/PVDF (Figure 2b) mixtures spectra. This is because the broad diffuse response of the PVDF amorphous phase is present, and the BaTiO_3_ major peaks (101) overlap with the PVDF α-phase peak at about 32° 2*θ* [14,37].

The XRD patterns of the multilayer films are shown in Figure 2c. The PVDF generally occurs in various phase modifications depending on chain orientation. The stable one is the α-phase. It is characterized by the appearance of about 18.5ons at about 18.5 and 20.2° 2*θ*, which is confirmed in diffractograms of all composite films. Further, the characteristic reflections originate from the *β*-cellulose present. The main characteristic of all polymer diffractograms is broad picks with low intensity, due to the high percentage of the amorphous phase and low inter-chain structural order of the polymer. A significant decrease in intensity was observed after the addition of the highly crystalline commercial BT. A gradual decrease in the intensity of all observed reflections, with the addition of mechanically activated BT, was noticed. The highest decrease is obvious for BT reflections, but it can be seen that reflections of the PVDF also decrease with increasing filler milling time. Since BT acts as a nucleation centre for PVDF crystallization, an increased number of the BT particles, due to applied mechanical treatment, as well as increased specific surface area and overall activity of it, obviously affect the crystallization behavior of PVDF, leading to the formation of the smaller crystallites [38]. It was shown that submicron-sized particles retard the movement of the polymer chain and impede the progress of crystallization [39]. The cellulose side of the multilayer film indicates the existence of pure cellulose without the presence of any secondary phase like lignin. Nominally pure cellulose is impossible to obtain from plants, but using bacteria for producing pure BNC is an often used method [40]. Further, it should be noticed that modification of the BNC with magnetite did not influence the phase stability of the original BNC. In the diffractograms of the composite, the broad, low-intensity peaks of the magnetite were also detected. Such appearance of the magnetite is due to the small crystallite size, which is a consequence of the controlled precipitation process.

Based on the obtained microstructural parameters (Table 3), prolonged milling time leads to a decrease in the crystallite size and an increase in the percentage of microstrain [41]. This is a result of the mechanical activation process caused by grinding, where the applied stress causes a reduction in the size of the grains and an increase in microstrain within the material. The decrease in crystal size can be observed by analyzing the powder diffraction pattern, where the broadening and significant decreases in reflection intensity were observed.

It is important to note that if the grinding process continues for a longer period of time, it can lead to the complete destruction of the crystal structure and the formation of an amorphous material [35]. This is because the mechanical stress applied during the grinding process can cause the crystals to break down into smaller particles, eventually leading to a loss of long-range order and the formation of an amorphous structure. Thus, it is crucial to control the duration of the grinding process in order to achieve the desired microstructure without causing severe damage to the material.

**Figure 3:**
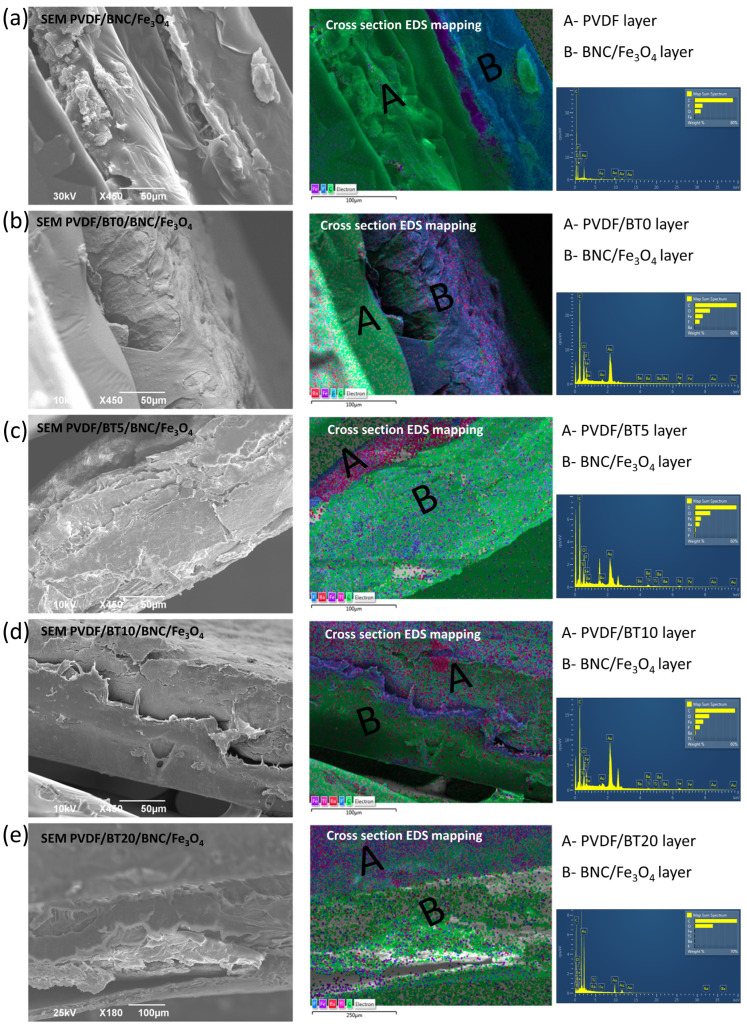
SEM and EDS of the samples (**a**) PVDF/BNC/Fe_3_O_4_ (cross section and corresponding EDS mapping); (**b**) PVDF/BT0/BNC/Fe_3_O_4_ (cross section and corresponding EDS mapping); (**c**) PVDF/BT5/BNC/Fe_3_O_4_ (cross section and corresponding EDS mapping); (**d**) PVDF/BT10/BNC/Fe_3_O_4_ (cross section and corresponding EDS mapping); and (**e**) PVDF/BT20/BNC/Fe_3_O_4_ (cross section and corresponding EDS mapping).

The micrographs of the composite sample are shown in Figure 3. For each of the five samples, two images are shown. The first image represents the cross section of the sample, while the second image shows the corresponding EDS mapping for that sample.

The BNC sides are characterized by a highly rough surface with a spherical Fe_3_O_4_ particle well-distributed overall surface. In contrast, the PVDF sides are much smoother and more uniform with elongated PVDF chains. The cross-sectional images show a visible difference between the layers but also indicate that the film layers are well connected. From the EDS mapping, it can be seen that Fe is mainly concentrated near the surface of the cellulose layers, while BT particles are well distributed in the PVDF matrix. Such occurrences can be connected to the preparation methods. The Fe_3_O_4_ was precipitated in the presence of solid BNC hydrogels in a reacted container. The synthesized particles could not penetrate deep into the BNC structure regardless of the ultrasound-assisted process. On the other hand, the PVDF-based layers were prepared by homogenous mixing of the BT and PVDF powders and afterward applying hot pressing to form this layer from a melt, which leads to better homogeneity.

By comparing the PVDF film with PVDF/BT films of different BT activation times, the following observations can be made. The value of the relative dielectric constant for pure PVDF at lower frequencies is around 10, which is confirmed in the literature [6,42].

**Figure 4:**
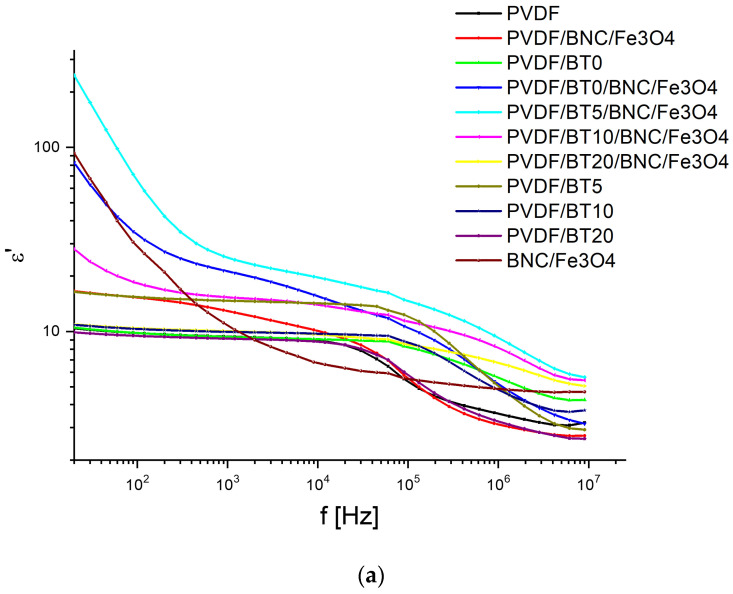

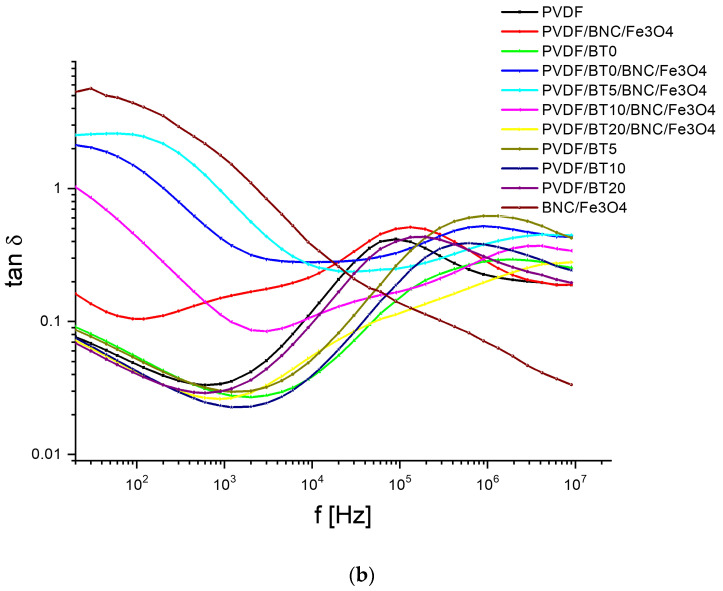
Results obtained experimentally using sinusoidal and triangular signals: (**a**) frequency dependence of relative dielectric constant (ε′), (**b**) frequency dependence of dielectric loss tangent (tan δ).

The value is quite stable up to 10 kHz, where there is a sudden drop due to a relaxation process [43]. In Figure 4b, the dipolar relaxation can be observed as a peak or maximum at around 90 kHz in the loss tangent spectrum. When comparing PVDF sample with com posites PVDF/BT0, PVDF/BT5, PVDF/BT10, and PVDF/BT20, the following can be noted. By introducing filler into the PVDF polymer matrix, the peaks of the dielectric loss tangent are shifted toward higher frequencies, and this is seen as a sudden drop in the epsilon spectrum compared to ε′ of pure PVDF, which is also confirmed in literature [43]. In Figure 4a, it can be seen that different activation times of the filler BT, at the same BT mass fraction of 5% in the PVDF/BT composite, have a different effect on the value of the ε′. For sample PVDF/BT5, the highest value of ε′ is observed, followed by sample PVDF/BT10, while sample PVDF/BT20 recorded a slight decrease in value at lower frequencies. As for sample PVDF/BT0, where BT particles are not activated, the pose ε′ value remains at lower frequencies, similar to pure PVDF.

To analyze the dielectric properties of the complex system and determine the impact of BNC Fe_3_O_4_, we compared PVDF/BT composites with different activation times to the corresponding bilayer samples of PVDF/BT (5,10,20) + BNC Fe_3_O_4_, i.e., samples PVDF/BT0/BNC/Fe_3_O, PVDF/BT5/BNC/Fe_3_O_4_, PVDF/BT10/BNC/Fe_3_O_4_, and PVDF/BT20/BNC/Fe_3_O_4_.

Observing the loss tangent for samples PVDF/BT0 and PVDF/BT0/BNC/Fe_3_O_4_, it can be seen that sample PVDF/BT0/BNC/Fe_3_O_4_ has higher tan δ values throughout the frequency range compared to sample PVDF/BT0, especially at lower frequencies, while they gradually decrease with increasing frequency. Moreover, regarding ε′, it is seen that the value for sample PVDF/BT0/BNC/Fe_3_O_4_ is significantly higher than the value for sample PVDF/BT0, especially at lower frequencies. As the frequency increases, these differences decrease and become similar. It is also noticeable that sample PVDF/BT0 has a fairly stable value of ε′ up to 100 kHz with a slight decrease in ε′ value.

The next comparative pair is samples PVDF/BT5/BNC/Fe_3_O_4_ and PVDF/BT5. Sample PVDF/BT5/BNC/Fe_3_O_4_ has significantly higher loss tangent at low and medium frequencies compared to sample PVDF/BT5, while at higher frequencies, these loss tangents are very similar. In the epsilon spectrum, it can be seen that the values for sample PVDF/BT5/BNC/Fe_3_O_4_ are high at low frequencies compared to sample PVDF/BT5, while these differences decrease with increasing frequency. Comparing samples PVDF/BT10/BNC/Fe_3_O_4_ and PVDF/BT10 shows a similar trend as the previous two pairs of samples. Regarding samples PVDF/BT20/BNC/Fe_3_O_4_ and PVDF/BT20, we see that the dielectric loss tangents for both samples have similar values, but at medium frequencies, starting from 1 kHz, the value of the dielectric loss tangent in sample PVDF/BT20/BNC/Fe_3_O_4_ is smaller than in sample PVDF/BT20. With increasing frequency, the losses in sample PVDF/BT20/BNC/Fe_3_O_4_ decrease compared to sample PVDF/BT20. As for ε′, it can be seen that both samples have stable values at lower frequencies, with sample PVDF/BT20/BNC/Fe_3_O_4_ having an 8% higher value than sample PVDF/BT20. In addition, sample PVDF/BT20/BNC/Fe_3_O_4_ has a more stable value of ε′ in a wider frequency range up to 100 kHz, while sample PVDF/BT20 experiences a sharp drop in epsilon value at 10 kHz.

The samples PVDF/BT0/BNC/Fe_3_O_4_, PVDF/BT5/BNC/Fe_3_O_4_, and PVDF/BT10/BNC/Fe_3_O_4_ have high loss tangent and an increase in relative dielectric constant in the lower frequency range, which can be explained by space charge or interface polarization that occurs due to the accumulation of charges at the interface between two different materials with different dielectric constant values. In the lower frequency range, sample PVDF/BT20/BNC/Fe_3_O_4_ has a relatively small increase in dielectric constant (about 5%) and in some sense stabilizes the dielectric constant in the entire measurement range compared to pure PVDF samples. Sample PVDF/BT20/BNC/Fe_3_O_4_ also shows a reduction in loss tangent compared to samples PVDF/BT0/BNC/Fe_3_O_4_, PVDF/BT5/BNC/Fe_3_O_4_, and PVDF/BT10/BNC/Fe_3_O_4_. The observed maximum of tan δ (Figure 4b) most probably originates from dipole orientation polarization which occurs due to the permanent electric dipole moment of fluorine and hydrogen substituents located on opposite sides of the polymer chain of PVDF perpendicular to the chain direction, also due to permanent dipole moment of cellulose (BNC) surface hydroxyl groups. These groups orientate in the direction of the external alternating electric field. The maximum of tan δ could give information about relaxation time (frequency at which tan δ has maximum). It can be seen that the maximum peaks shift toward higher frequencies with increasing activation time. There is a disturbance in the local electric field at the interface between the polymer and activated BaTiO3 particles, causing a change in relaxation time or frequency. 

Based on the improved dielectric properties of sample PVDF/BT20/BNC/Fe_3_O_4_, it could potentially be applied in various fields such as electronics, energy storage, and sensing applications. For example, it could be used in the development of high-performance capacitors, sensors, and transducers. The reduced losses and stable dielectric constant make it a promising material for use in high-frequency applications.

## 4. Conclusions

The study aimed to create a versatile multilayer PVDF-based composite for intelligent packaging. Constituted by PVDF/BaTiO_3_ and Fe_3_O_4_-modified bacterial nanocellulose (BNC) hydrogels, these layers were merged via hot pressing. Structural, morphological, and dielectric properties of the produced multilayer film were investigated. EDS mapping affirmed uniform BT distribution within the polymer layer, while Fe_3_O_4_ particles concentrate at the cellulose surface. The impact of BT presence and duration of the mechanical activation on the relative dielectric permittivity of the composites was assessed. The addition of BT fillers into the PVDF matrix shifts the dipolar relaxation peak of loss tangent to higher frequencies. Optimal results were achieved in the multilayer composite (PVDF/BT20/BNC/Fe_3_O_4_) with extended BT activation time, displaying the best dielectric performance and loss tangent reduction. Monolayered composite (PVDF/BT20) found stable relative dielectric permittivity at lower and mid frequencies. Multilayered PVDF/BT20/BNC/Fe_3_O_4_ exhibited an 8% higher value of Ɛr. Notably, this sample also displayed superior stability in the relative dielectric permittivity across a broader frequency range (up to 100 kHz).

## Figures and Tables

**Table 1:** Tabular representation of particle distribution and sizes as a function of volume fraction for samples BT0, BT5, BT10, and BT20.

	BT0	BT5	BT10	BT20
**d(0.1) (μm)**	0.644	0.457	0.556	0.485
**d(0.5) (μm)**	1.458	1.115	1.336	1.244
**d(0.9) (μm)**	10.710	11.421	11.727	11.718
**Span**	6.904	9.831	8.361	9.026

**Table 2:** Results of crystallite size display and their stress values for powder BT samples.

Sample Name	*BT*		*BT5*		*BT10*		*BT20*	
Phase Name	Crystallite Size (Å)	Strain (%)	Crystallite Size (Å)	Strain (%)	Crystallite Size (Å)	Strain (%)	Crystallite Size (Å)	Strain (%)
**Perovskite** **BaTiO_3_**	491 (2)	0.000000	219 (9)	0.39 (7)	183 (9)	0.57 (8)	153 (7)	0.64 (5)

**Table 3:** Microstructural parameters of the multi-layered film samples.

Sample Name	*PVDF/BNC/Fe_3_O_4_*		*PVDF/BT0/BNC/Fe_3_O_4_*		*PVDF/BT5/BNC/Fe_3_O_4_*		*PVDF/BT10/BNC/Fe_3_O_4_*		*PVDF/BT20/BNC/Fe_3_O_4_*	
Phase Name	Crystallite Size (Å)	Strai n (%)	Crystallite Size (Å)	Strain (%)	Crystallite Size Å)	Strain (%)	Crystallite Size (Å)	Strain (%)	Crystallite Size (Å)	Strain (%)
α-PVDF	72 (5)	0.40 (3)	72 (7)	0.50 (4)	36 (3)	0.43 (5)	35 (2)	0.52 (3)	24.7 (4)	0.0000
Magnetite Fe_3_O_4_	85 (3)	0.72 (2)	87 (4)	0.98 (3)	75 (6)	0.12 (4)	63 (4)	0.23 (4)	25.5 (2)	1.23 (6)
Cellulose I-β	77 (9)	0.71 (4)	68 (6)	0.74 (5)	42 (3)	0.73 (6)	41 (6)	0.75 (5)		
Perovskite Ba-TiO_3_			469 (9)	0.15 (6)	273 (22)	0.49 (5)	174 (39)	0.51 (4)	78 (4)	0.71 (3)

## Data Availability

The raw/processed data required to reproduce these findings cannot be shared at this time as the data also form part of an ongoing study.
